# An optical keypad lock with high resettability based on a quantum dot–porphyrin FRET nanodevice†

**DOI:** 10.1039/d3na00030c

**Published:** 2023-05-08

**Authors:** Peng Shen, Yuqian Liu, Xiaojun Qu, Mingsong Zhu, Ting Huang, Qingjiang Sun

**Affiliations:** a State Key Laboratory of Bioelectronics, School of Biological Science & Medical Engineering, Southeast University Nanjing 210096 China sunqj@seu.edu.cn; b Jiangsu Co-Innovation Center of Efficient Processing and Utilization of Forest Resources, Joint International Research Lab of Lignocellulosic Functional Materials, College of Light Industry and Food Engineering, Nanjing Forestry University Nanjing 210037 China

## Abstract

Due to their appealing properties, nanomaterials have become ideal candidates for the implementation of computing systems. Herein, an optical keypad lock based on a Förster resonance energy transfer (FRET) nanodevice is developed. The nanodevice is composed of a green-emission quantum dot with a thick silica shell (gQD@SiO_2_) and peripheric blue-emission quantum dots with ultrathin silica spacer (bQD@SiO_2_), on which 5,10,15,20-tetrakis(4-sulfophenyl)porphyrin (TSPP) is covalently linked. The nanodevice outputs dual emission-based ratiometric fluorescence, depending on the FRET efficiency of bQD–porphyrin pairs, which is highly sensitive to the metalation of TSPP: values are 59.7%, 44.8%, and 10.1% for bQD–Zn(ii)TSPP, bQD–TSPP, and bQD–Fe(iii)TSPP pairs, respectively. As such, by using the competitive chelation-induced transmetalation of TSPP, the nanodevice is capable of implementing a 3-input keypad lock that is unlocked only by the correct input order of Zn(ii) chelator, iron ions, and UV light. Interestingly, the reversible transmetalation of TSPP permits the reset (lock) operation of the keypad lock with the correct input order of ascorbic acid, Zn(ii), and UV light. Application of the nanodevice is exemplified by the construction of paper and cellular keypad locks, respectively, both of which feature signal readability and/or high resettability, showing high potential for personal information identification and bio-encryption applications.

## Abstract 2

A QD-FRET nanodevice based 3-input keypad lock is demonstrated to provide a low-cost but robust way toward next-generation security systems for personal information identification and bio-encryption applications.

## Graphical abstract

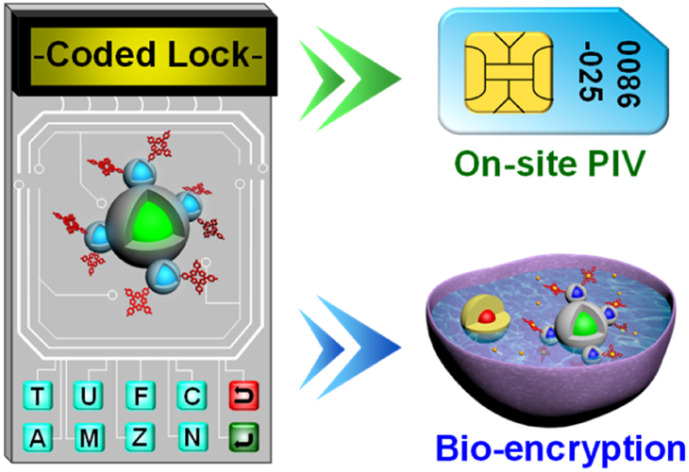

## Introduction

Molecular computing, which mimics the functions of the logic gates that constitute the basis of digital information processing, has become an important research field within modern unconventional computing.^1–3^ Over the past two decades, basic logic gates (AND, OR, INHIBIT, *etc.*)^4,5^ and advanced logic devices (half-adder/half-subtractor, full-adder/full-subtractor, multiplexer/demultiplexer, encoder/decoder, and multiple-cascade logic circuits)^6–12^ have been constructed for information processing or storage. Given the extensive, ever-growing research on molecular computing, it is important to develop a molecular security system that prevents illegal data invasion. The molecular keypad lock represents a new approach to information protection at the molecular level. What complicates a keypad lock device over other logic devices lies in the fact that it is obtained *via* the integration of several AND logic gates and their output signal is dependent not only on receiving the proper combination of inputs, but also on introducing them in the correct order.^13–15^

By cascading photochemical reactions, researchers have devised molecular keypad locks that draw on metastable situations. Since Margulies *et al.* reported the first molecular keypad lock using a fluorescein-linker-pyrene assembly,^13^ a number of unimolecular systems with various inputs, such as cations, anions, or light, have been reported sporadically.^16–19^ Currently, improving the complexity of a keypad lock device by increasing the number of inputs or outputs so as to enhance the device security level is on the front burner.^20,21^ From this viewpoint, biomolecular keypad lock devices that are based on concatenation of enzymatic catalytic reaction,^22,23^ biomolecular recognition,^24–26^ or even cell signaling transduction^27,28^ have been elegantly designed. It is possible to increase the number of password entry bits simply by introducing more inputs. Integration of (bio)molecular receptors with micro- or nanomaterials such as gold nanoclusters,^29^ carbon dots,^30,31^ or silica mesoporous microspheres^32^ has opened new dimensions of keypad lock development. Their robust physical and chemical properties are available while the nanomaterials are used as substrates or signal transduction units. For example, Zhu *et al.*^33^ and Chen *et al.*^34^ have developed keypad lock devices on substrates of silver microspheres and magnetic beads, respectively, by harnessing sequential DNA hybridization reactions. The microspheres/beads facilitate the separation of devices from incorrect inputs, offering input order dependence. Simultaneously, the ease of device separation provides them with resettability by virtue of DNA denaturation or competitive hybridization. Huang *et al.*^35^ devised a keypad lock that used gold nanoparticles as the signal transduction unit. The keypad lock exhibited noticeable colour change upon the correct “password” being provided, featuring signal-read easiness.

Semiconductor quantum dots (QDs) provide a plethora of unique photophysical properties, such as high quantum yields, narrow-band emission, broad-band extinction, and resistance to photo-bleaching, which are of great significance for construction of Förster resonance energy transfer (FRET) configurations.^36^ By tuning the FRET efficiency *via* either changing the donor–acceptor distance or adjusting the resonance effect in the biochemical or chemical reactions, QD-FRET systems are vastly used for bioimaging and chemical/bio-sensing.^37–40^ However, the use of QD-FRET systems to construct logic devices for computational applications is far less explored. Relying on dynamic assembly and disassembly of donors and acceptors, Claussen *et al.*^41^ reported a QD photonic logic device that supported the logic operations of OR, AND, INHIBIT, XOR, NOR, and NAND gates. He *et al.*^42^ reported a DNA-programmed QD-FRET system and realized a complete set of seven elementary logic gates (OR, AND, NOR, NAND, INH, XOR, and XNOR). Although data processing was achieved using proper gate combinations in these studies, no keypad lock device has been implemented for data protection thus far. Besides, it is highly desirable to implement a logic device with the static QD-FRET system, in which the acceptor is closely bonded to the QD and the FRET efficiency is controlled by regulating the resonance effect of the donor–acceptor pair. Such an architecture might give rise to highly reproducible FRET tuning only by changing the acceptor absorption instead of the system reconfiguration.

**Scheme 1:**
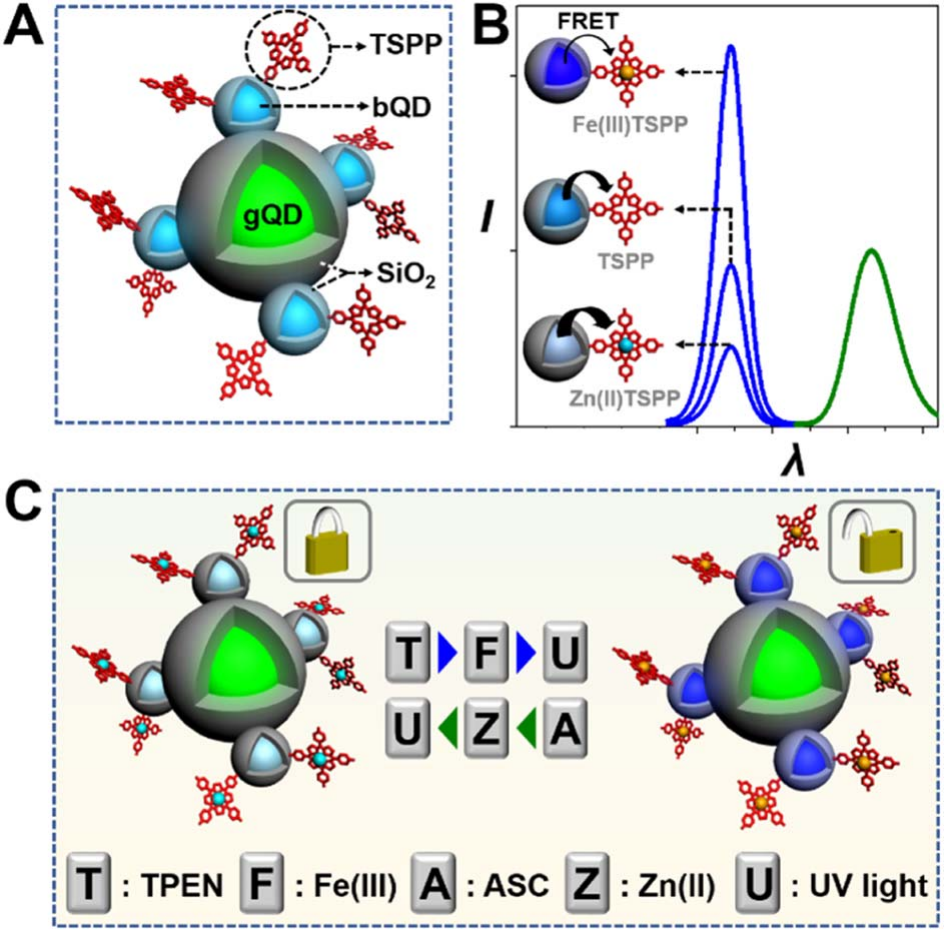
(A) Structure of the QD–TSPP FRET nanodevice. (B) Fluorescence spectra of the nanodevice with various FRET acceptors: Fe(iii)TSPP, TSPP, and Zn(ii)TSPP. (C) Illustration of the unlock and lock operations of the keypad lock device.

This work reports an optical keypad lock device based on a reversibly adjustable QD-FRET system. The nanodevice comprises an “always-on” fluorophore, silica coated green-emission QD (gQD@SiO_2_), the FRET donor, ultrathin silica coated blue-emission QD (bQD@SiO_2_), and the FRET acceptor, 5,10,15,20-tetrakis(4-sulfophenyl)porphyrin (TSPP). As shown in Scheme 1(A), TSPP is covalently linked to the bQD@SiO_2_ to ensure the FRET at the fixed distance. The bQD@SiO_2_–TSPP is assembled on gQD@SiO_2_ to form a “core-satellite” structure. In such a static QD–TSPP FRET nanodevice, the FRET efficiency is determined by the absorption wavelength shift when TSPP complexes with various metal ions. This leads to the emission intensity variations of bQD@SiO_2_ and further gives rise to the ratiometric fluorescence, as well as emission colour change of the nanodevice. The blue-to-green emission intensity ratios (*I*_424_/*I*_517_) are 1 : 2.2 and 2.2 : 1 when Zn(ii) and Fe(iii), respectively, are incorporated into the TSPP nucleus (Scheme 1(B)). From the perspective of a keypad lock, the aforementioned emission intensity ratios are defined as the “CLOSED” and “OPEN” states, respectively. By harnessing the principle of competitive metal ion chelation, the keypad lock can be unlocked upon *N*,*N*,*N*′,*N*′-tetramethylethylenediamine (TPEN, T), Fe(iii) (F), and UV light (U) are added sequentially, as shown in Scheme 1(C). In this process, Zn(ii) to Fe(iii) transmetalation of TSPP occurs and the fluorescence of bQD@SiO_2_ recovers due to suppression of the FRET efficiency. Importantly, based on the reversible transmetalation process of TSPP, the nanodevice with the “OPEN” state can be locked again upon sequential addition of ascorbic acid (ASC, A), Zn(ii) (Z), and UV light. Featuring excellent resettability, easy signal readability, and high stability, as a proof-of-concept, the nanodevice is further challenged to construct a paper keypad lock for personal information verification (PIV) and a cellular keypad lock for bio-encryption.

## Experimental

### Materials and reagents

Tetraethyl orthosilicate (TEOS, 99.999%), 3-(trimethoxysilyl)-1-propanamine (APTMS, 97%), (3-mercaptopropyl)trimethoxysilane (MPTMS, 95%), TSPP (≥95.0%), TPEN (≥98.0%), ZnCl_2_ (99.99%), and ammonia solution (99.99%) were supplied by Sigma-Aldrich. Ascorbic acid (99%) was purchased from J&K. SOCl_2_ (99.5%), imidazole (99.5%), NaNO_2_ (99%), FeCl_3_ (99.9%), and chitosan (≥95% deacetylated) were obtained from Aladdin. Phosphoric acid (analytical reagent), nitric acid (analytical reagent), and cellulose paper were purchased from Wanqing Chemical (Nanjing, China). Pure water (18.2 MΩ cm^−1^) was obtained from a Pall Cascade AN system.

### Logic operations of the keypad lock

The keypad lock states were defined as “CLOSED” and “OPEN” states when the FRET acceptors were Zn(ii)TSPP and Fe(iii)TSPP, respectively. For the unlock operation, 100 μL of TPEN solution (4 μM) and 100 μL of FeCl_3_ solution (10 μM, in the coexistence of 10 μM of FeCl_2_·7H_2_O) were sequentially added into 200 μL of nanodevice solution (50 nM). Finally, the solution was irradiated using a Xe lamp (400 nm) or UV lamp (365 nm) to record the fluorescence spectra or read the emission colour. The lock operation was performed by sequentially inputting 100 μL of ASC solution (4 μM), 100 μL of ZnCl_2_ solution (10 μM, containing 120 mM of imidazole), and UV light to the nanodevice solution. To reveal the input sequence dependence of the output, the chemical inputs and UV light were added in random orders and the fluorescence spectra were measured during UV irradiation. Each chemical input was followed by a 40 min standing to guarantee sufficient TSPP metalation or demetalation. In addition, a centrifugation (3000 rpm, 5 min) process was performed after each complete unlock or lock operation to separate the nanodevice from historical inputs. The precipitated nanodevice was re-dispersed in 200 μL of PBS buffer and exposed to another logic operation.

### Demonstration of a paper keypad lock

In order to simulate the PIV cards, we constructed a paper keypad lock *via* layer-by-layer assembly of the nanodevice on a paper substrate. The cellulose paper was cut into subscriber identification module (SIM) card-shaped strips (2.5 × 1.5 cm), and oxidized using a mixture of NaNO_2_, phosphoric acid, and nitric acid.^43^ Except for a square area (0.8 × 0.8 cm), the strip was hydrophobically processed with wax. Afterward, the surface charge of the cellulose paper was inversed by dropping 80 μL of chitosan solution (1 mg mL^−1^ in 0.01 M acetic acid solution) onto the hydrophilic area, followed by three times washing with pure water to remove excess chitosan. After natural drying, 40 μL of nanodevice solution with a concentration of 100 nM was dropped onto the chitosan-containing paper substrate. The paper keypad lock was fabricated after thorough volatilization of water. In the simulated PIV experiment, each chemical input (40 μL) was added to the paper keypad lock or UV light was inputted by the UV lamp. The concentrations of the chemicals were consistent with those used to unlock and lock the keypad lock in the homogeneous phase. The results were imaged with a smartphone. The paper keypad lock was recycled by simply immersing it in pure water for 10 min to remove the chemical inputs.

### Demonstration of a cellular keypad lock

The cellular keypad lock was demonstrated with the HeLa cell as a model. HeLa cells were cultured in 10% FBS-supplemented Dulbecco's Modified Eagle Medium at 37 °C in a 5% CO_2_ humidified atmosphere. The keypad lock treated cells were obtained upon spiking 100 nM of nanodevice into the culture medium for 4 h. The cells were further fixed with paraformaldehyde after being washed three times with PBS buffer to remove the unendocytosed nanodevice. The logic operations of the cellular encryption device were performed by sequentially introducing 200 μL of chemical inputs (consistent in concentration with the extracellular experiments) or UV laser into the cell culture microporous plates. Fluorescence images of cellular encryption devices were taken and analyzed using ImageJ software for Windows (Version: 1.52v).

## Results and discussion

### Assembly of the “core-satellite” nanodevice

**Fig. 1:**
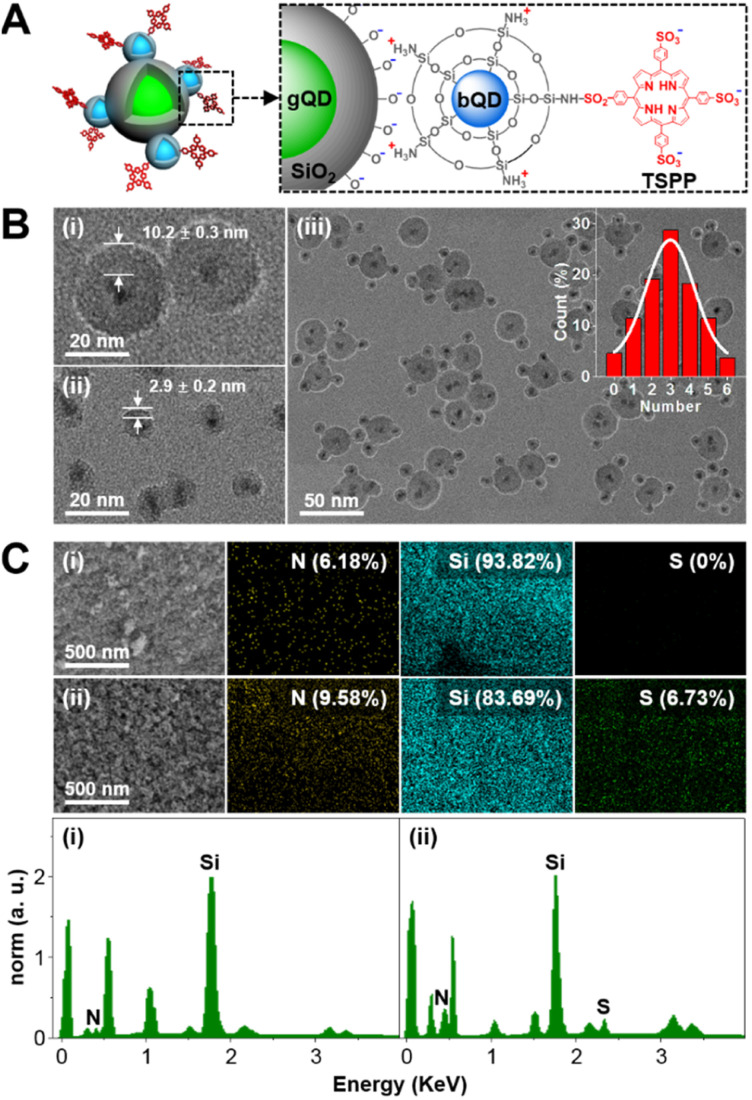
(A) Illustration of “core-satellite” structured gQD@SiO_2_/bQD@SiO_2_–TSPP nanodevice. (B) TEM images of (i) gQD@SiO_2_, (ii) bQD@SiO_2_–TSPP and (iii) gQD@SiO_2_/bQD@SiO_2_–TSPP. (C) SEM images, EDX mapping and EDX spectra of (i) gQD@SiO_2_/bQD@SiO_2_ and (ii) gQD@SiO_2_/bQD@SiO_2_–TSPP.

With the purpose of ratiometric fluorescence output, as well as easy separation of the nanodevice, bQD@SiO_2_–TSPP was assembled onto larger-sized gQD@SiO_2_ (Fig. 1A). Both bQD@SiO_2_–TSPP and gQD@SiO_2_ were prepared by the QD silanization procedure. Considering the significant influence of the acceptor number on FRET efficiency, the TSPP density on bQD@SiO_2_ was adjusted by controlling the molar ratio of APTMS to APTMS–TSPP in the silanization process. The fluorescence of bQD is quenched to 48.6% with an APTMS to APTMS–TSPP ratio of 6 : 1, which is regarded as the optimized ratio for the preparation of bQD@SiO_2_–TSPP (Fig. S1 in the ESI†). The concentration of bQDs can be calculated according to the absorption spectra (Fig. S2 in the ESI†). Therefore, approximately 1.8 TSPP molecules are estimated to be modified onto the surface of one bQD@SiO_2_ under such a APTMS to APTMS–TSPP ratio. The thickness of SiO_2_ coated on gQD and bQD was tuned effectively by adjusting the quantities of silane reagents. Fig. 1B shows TEM images of gQD@SiO_2_, bQD@SiO_2_–TSPP, and the nanodevice. The SiO_2_ shell on gQD is about 10.2 nm-thick and can effectively prevent gQD fluorescence quenching from possible electron or energy transfer, while a 2.9 nm-thick SiO_2_ shell is coated onto bQD, which allows efficient FRET from bQD donor to TSPP acceptor. Multiple bQD@SiO_2_–TSPP nanoparticles are electrostatically absorbed onto the gQD@SiO_2_ surface, forming the “core-satellite” nanodevice. The statistical result (from 50 nanodevices) reveals that on average ∼3 bQD@SiO_2_ nanoparticles are assembled on the surface of one gQD@SiO_2_. The assembly is verified by the zeta potential measurements (Fig. S3 in the ESI†). Zeta potentials are −51.4 and 33.2 mV for silanol group-terminated gQD@SiO_2_ and amino group-dominated bQD@SiO_2_–TSPP, respectively. For the assembled gQD@SiO_2_/bQD@SiO_2_–TSPP nanodevice, the potential shifts to −15.8 mV. The composition of the nanodevice was analysed by energy dispersive X-ray spectroscopy (EDX). As can be seen in Fig. 1C, the EDX mapping and spectra clearly show that Si (band at 1.74 keV) is the main composition for both gQD@SiO_2_/bQD@SiO_2_ and gQD@SiO_2_/bQD@SiO_2_–TSPP. The slight emergence of N (band at 0.39 keV) signal in gQD@SiO_2_/bQD@SiO_2_ is attributed to the aminated silane agent APTMS. For gQD@SiO_2_/bQD@SiO_2_–TSPP, the signal intensities as well as the atomic concentrations of N and S (band at 2.31 keV) increase compared to gQD@SiO_2_/bQD@SiO_2_. The result indicates the successful modification of TSPP on bQD@SiO_2_, as well as the assembly of bQD@SiO_2_–TSPP on the surface of gQD@SiO_2_. The chemical bonding details were further revealed by Fourier transform infrared (FT-IR) spectroscopy (Fig. S4 in the ESI†). The peaks at 1095 cm^−1^, 1419 cm^−1^, and 2890 cm^−1^ are originated from the vibrations of Si–O–Si bond in silica, C–N bond in TSPP, and –CH_2_– linker in APTMS, respectively.

### Unlock of the keypad lock

**Fig. 2:**
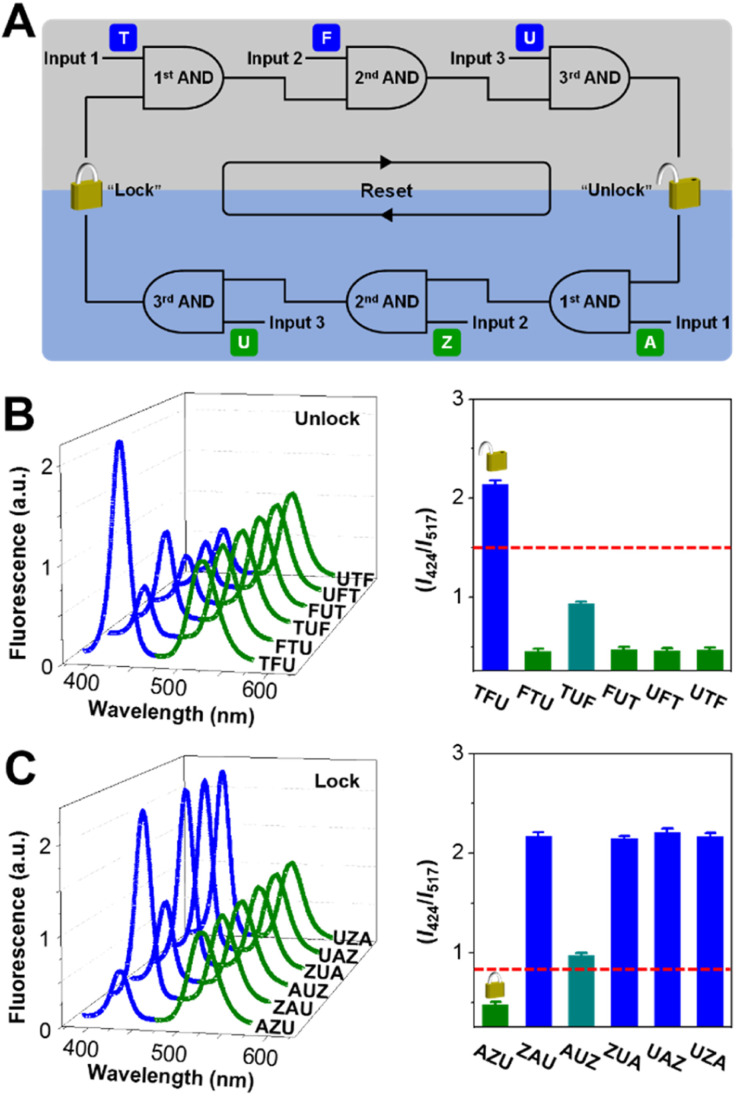
(A) Representation of the keypad lock as a network of concatenated AND gates. Nanodevice fluorescence spectra and corresponding fluorescence ratio bars for various input sequences in (B) the unlock operation and (C) the lock operation.

The unlock operation starts with the keypad lock in the “CLOSED” state, in which Zn(ii) is incorporated into the TSPP nucleus and the outputted *I*_424_/*I*_517_ is 1 : 2.2. As shown in Fig. 2A, the unlock operation is presented as a network composed of three concatenated AND gates, in which the output of one gate serves as an input for a downstream gate. In the 1st AND gate, input of TPEN (T) leads to the demetallation of Zn(ii)TSPP. The output of the 1st AND gate then complexes with the inputted Fe(iii) (F) under the catalysis of Fe(ii) and activates the 2nd AND gate. Subsequently, UV light (the input U) and the output of the 2nd gate trigger the 3rd gate to output an unlocked nanodevice with a greatly improved *I*_424_/*I*_517_. We have systematically investigated the absorption properties of TSPP for sake of the feasibility of unlocking operation and optimizing the standing time between the input intervals. As shown in Fig. S5 in the ESI,† the Soret-band absorption peak of TSPP blue-shifts upon continuous addition of TPEN and Fe(iii), with 40 min equilibrium time, indicative of Zn(ii) to Fe(iii) transmetalation of TSPP. The chelation state of TSPP determines the fluorescence property of the nanodevice, as shown in Fig. 2B. The green fluorescence is observed to be consistent for all six possible input orders: TFU, FTU, TUF, FUT, UTF, and UFT. For the blue fluorescence, variations are negligible upon the inputs are added following the orders of FTU, FUT, UTF, and UFT. However, for the input combinations of TUF and TFU, the blue fluorescence is slightly and fully recovered. Accordingly, the outputted *I*_424_/*I*_517_ values are 0.9 for TUF, 2.2 for TFU, and 0.5 for other input orders. When the *I*_424_/*I*_517_ value of 1.5 is defined as the threshold of “OPEN” state, the keypad lock can be unlocked only by introducing the inputs in the correct order: TFU. Otherwise, the unlock operation fails.

### Lock of the keypad lock

Resettability is a key factor for keypad lock devices from the viewpoint of practical application. For those resettable molecular keypad lock devices, the “CLOSED” state could be reached again *via* physical/chemical treatments, however, in a non-logical manner. Herein, we explore the unusual reset (lock) operation of the proposed keypad lock: the nanodevice is switched back to the “CLOSED” state only if the resetting elements are inputted in the correct order. As shown in Fig. 2A, the circuit indicates that the nanodevice can be locked *via* the operation of three concatenated AND gates with sequential inputs of ASC (A), Zn(ii) (Z), and UV light. The lock operation of the keypad lock is based on Fe(iii) to Zn(ii) transmetalation of TSPP, which is demonstrated by successive red-shift of Soret-band absorption of TSPP as shown in Fig. S6 in the ESI.† As it is Fe(iii) reductant as well as the iron complexant,^44^ ASC can induce the demetalation of Fe(iii)TSPP. Re-formation of Zn(ii)TSPP then occurs when Zn(ii) is inputted. As shown in Fig. 2C, the AZU input combination quenches the blue fluorescence of the nanodevice by 74.8%. The quenching is 54.3% for the combination of ZAU and no obvious fluorescence quenching is observed for other input combinations. Accordingly, the blue/green fluorescence ratios vary with the input combinations. Only the AZU combination decreases the *I*_424_/*I*_517_ value below the set threshold of 0.8, switching the keypad lock to its “CLOSED” state.

### Repetitive unlock-lock operations

**Fig. 3:**
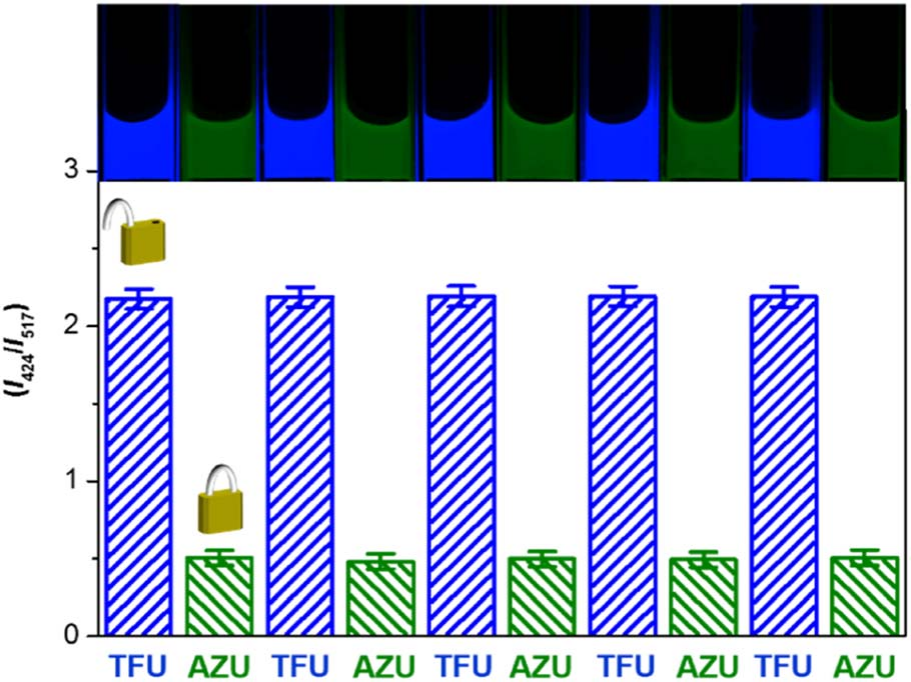
Fluorescence ratio histogram of keypad lock unlock-lock cycles triggered by TFU and AZU input combinations. Inset images show the emission colour of the keypad lock.

The unlock and lock operations of the keypad lock nanodevice encouraged us to investigate its ability to implement repetitive unlock-lock operations, as is the case for electronic encryption devices. After each unlock or lock operation, the nanodevice was separated from the chemical inputs by centrifugation and re-dispersed in PBS buffer for being operated by the new input string. As shown in Fig. 3 and S7 in the ESI,† the fluorescence signals remain nearly identical for five repeated unlock-lock cycles. The standard derivations are 0.8 and 0.9% for unlock and lock operations, respectively. Blue emission is switched “On” by TFU and “Off” by AZU. Owing to dual emission-based ratiometric fluorescence output, the nanodevice exhibits distinguishable colour changes that can be detected by the naked eye during the operation cycles, featuring signal-read easiness. As shown in the inset of Fig. 3, the nanodevice turns blue in the “OPEN” state and green in the “CLOSED” state. These results clearly reveal that the nanodevice is capable of repetitive unlock-lock operations, which is benefited from its separation easiness and high chemical/optical stability.

### Absorption wavelength shift controlled FRET

**Fig. 4:**
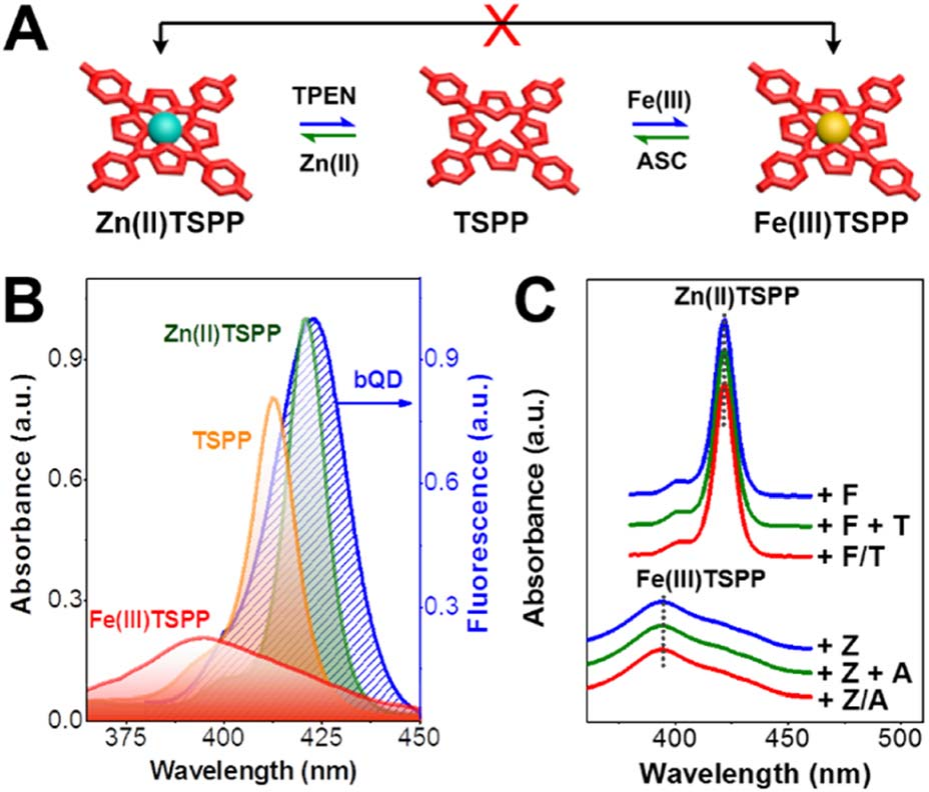
(A) Illustration of reversible transmetalation of TSPP based on the competitive metal chelation reaction. (B) Spectral overlaps between bQD emission and absorptions of Zn(ii)TSPP, TSPP, and Fe(iii)TSPP. (C) Absorption spectra of Zn(ii)TSPP and Fe(iii) TSPP under various chemical input sequence.

After the implementation of cycled unlock and lock operations, we investigated the working principle of the keypad lock nanodevice. The FRET between bQD and TSPP is regarded as the primary mechanism that accounts for the change in blue fluorescence. In the unlock/lock operations, transformation occurs among Zn(ii)TSPP, TSPP, and Fe(iii)TSPP (Fig. 4A). In the context of a constant donor–acceptor distance (*r*), which is 5.9 nm based on the radius of a bQD (Fig. S8 in the ESI†) together with the thickness of ultrathin silica shell, the FRET efficiency is determined by spectral overlap between the emission spectrum of bQD and the absorption spectra of porphyrin/metalloporphyrins. In the nanodevices, bQD@SiO_2_ possesses consistent quantum yield (QY_D_) of 51% (Table 1). According to the equations for calculating the FRET parameters,^40^ the spectral overlap integral (*J*(*λ*)) has thus become the exclusive factor that impacts the Förster radius (*R*_0_), and further the FRET efficiency. As shown in Fig. 4B, the Soret-band absorption peaks locate at 393, 412, and 422 nm for Fe(iii)TSPP, TSPP, and Zn(ii)TSPP, respectively. Moreover, the extinction coefficient remarkably increases along with the successive transformation from Fe(iii)TSPP to TSPP, and then Zn(ii)TSPP. Both the absorption peak wavelength and the extinction coefficient for porphyrins remain the same as the findings in previous reports.^45–47^ Therefore, the spectral overlap integral follows the order *J*(*λ*)^bQD–Zn(ii)TSPP^ > *J*(*λ*)^bQD–TSPP^ > *J*(*λ*)^bQD–Fe(iii)TSPP^, which leads to a gradually shortened Förster radius (*R*_0_) in the Zn(ii) to Fe(iii) transmetalation process. Relying on the *r*^−6^ dependence of FRET efficiency, the rates are 59.7% for bQD–Zn(ii)TSPP, 44.8% for bQD–TSPP, and 10.1% for bQD–Fe(iii)TSPP (Table 1). Accordingly, fluorescence ratiometry of the nanodevice is adjusted during the unlock-lock operations.

**Table 1:** Comparison of FRET parameters for bQD–Zn(ii)TSPP, bQD–TSPP and bQD–Fe(iii)TSPP

Acceptors	QY_D_^a^ (%)	*λ* (nm)	*ε* (M^−1^ cm^−1^)	*J*(*λ*) (M^−1^ cm^−1^ nm^4^)	*R*_0_ b (nm)	*r* (nm)	*E* c (%)
Zn(ii)TSPP	51	422	6.4 × 10^5^	1.3 × 10^13^	6.3	5.9	59.7
TSPP	51	413	4.6 × 10^5^	3.8 × 10^12^	5.7	5.9	44.8
Fe(iii)TSPP	51	393	1.3 × 10^5^	5.2 × 10^11^	4.1	5.9	10.1

a The quantum yield of bQD@SiO_2_ is calculated using diphenylanthracene as the standard (QY = 91% in ethanol).

b Calculated according to the equation: 
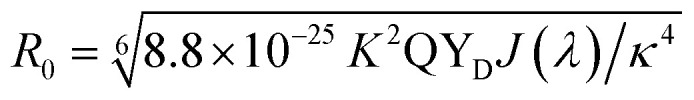
, where *K*^2^ is 2/3 for two randomly oriented dipoles in solution, *k* is the refractive index of the medium (1.33 for water), *J*(*λ*) is the spectral overlap integral.

c Calculated based on the equation: *E* = *R*_0_^6^/(*R*_0_^6^ + *r*^6^).

The correlation of the absorption wavelength shift of TSPP with the correct input order is fundamental to the keypad lock. To reveal the input order dependence, we measured the Soret-band absorption of Zn(ii)TSPP and Fe(iii)TSPP for varying input combinations. As shown in Fig. 4C, the absorption spectrum of Zn(ii)TSPP remains unchanged when: only Fe(iii) is added, TPEN is added after Fe(iii), or TPEN and Fe(iii) are added simultaneously. This is attributed to the competitive complexation between TPEN and TSPP toward Fe(ii), which plays the role as catalyst for the formation of Fe(iii)TSPP against Zn(ii). The dissociation constant of Zn(ii) and TSPP (*K*^Zn(ii)TSPP^_d_) is as high as 10^−6^ M.^48^ The values for *K*^Zn(ii)TSPP^_d_ and *K*^Zn(ii)TPEN^_d_ are at the levels of 10^−15^ M and 10^−14^ M, respectively.^49,50^ Due to the high *K*^Zn(ii)TSPP^_d_, Fe(ii) cannot replace Zn(ii) on the nucleus of Zn(ii)TSPP. When iron ions are inputted ahead of or together with TPEN, TPEN preferentially complexes with Fe(ii) by forming a highly stable Fe(ii)TPEN complex. Only excess of TPEN can induce demetalation of Zn(ii)TSPP. Similarly, the absorption spectrum of Fe(iii)TSPP remains constant except that ASC and Zn(ii) are added sequentially. This is also attributed to the competitive formation of Zn(ii)ASC and Fe(ii)ASC complexes.^51^

### Paper keypad lock for PIV demonstration

**Fig. 5:**
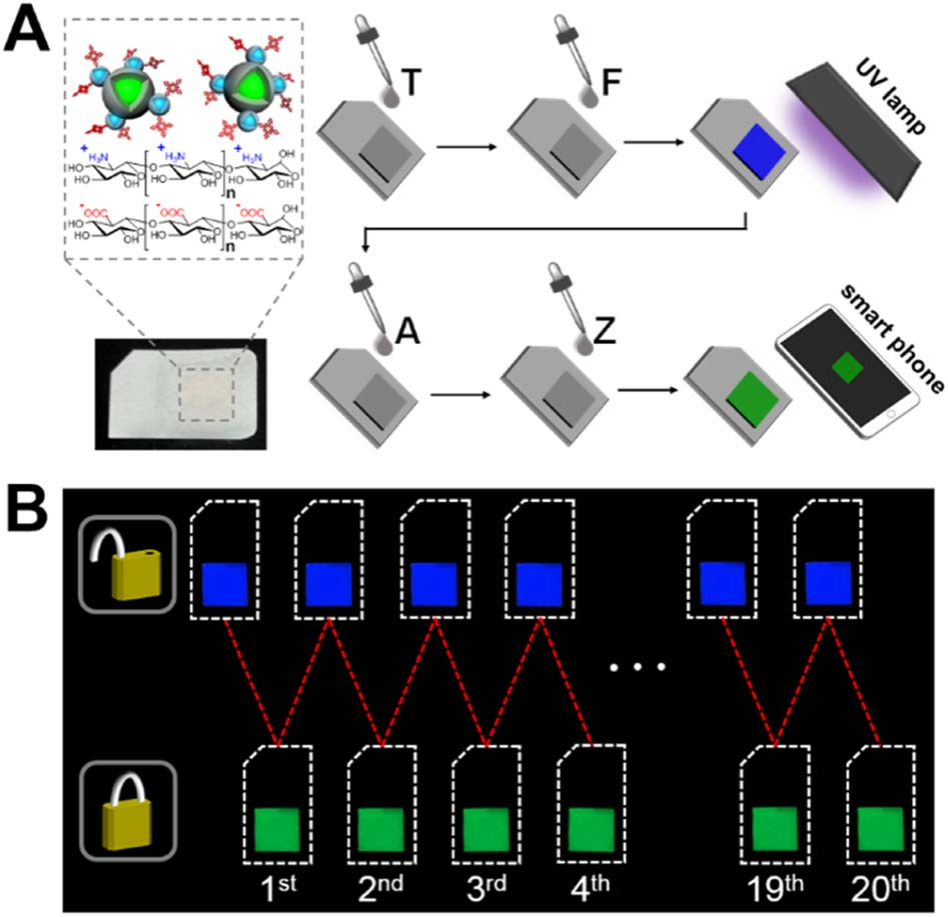
(A) Structure and operation protocol of paper keypad lock. (B) Fluorescent images of the paper keypad lock during repetitive unlock-lock operations.

The stability, resettability, and signal readability of the nanodevice-based keypad lock allow us to further explore a portable encryption system that simulates a PIV card. Cellulose paper is an ideal substrate for loading the nanodevice due to its advantages such as low cost and ease of modification. As shown in Fig. 5A, the nanodevice was immobilized on the hydrophilic area of SIM card-shaped paper strips *via* a layer-by-layer assembly strategy. Paper keypad lock operations were implemented by introducing the inputs onto its surface, followed by imaging the outputs with a smartphone. As shown in Fig. 5B, the paper keypad lock exhibits bright blue emission in the “OPEN” state when TFU is inputted, and bright green emission in the “CLOSED” state in response to AZU. This is consistent with the results from the keypad lock in the homogeneous solution. Other input combinations result in unchanged or slightly changed emission colours of the paper keypad lock (Fig. S9 in the ESI†), signifying failure to pass the verification. In addition, the stable fluorescent outputs for up to 20 repeated unlock-lock operations indicate that the paper keypad lock is reusable, revealing high potential for PIV application.

### Cellular keypad lock for bio-encryption

**Fig. 6:**
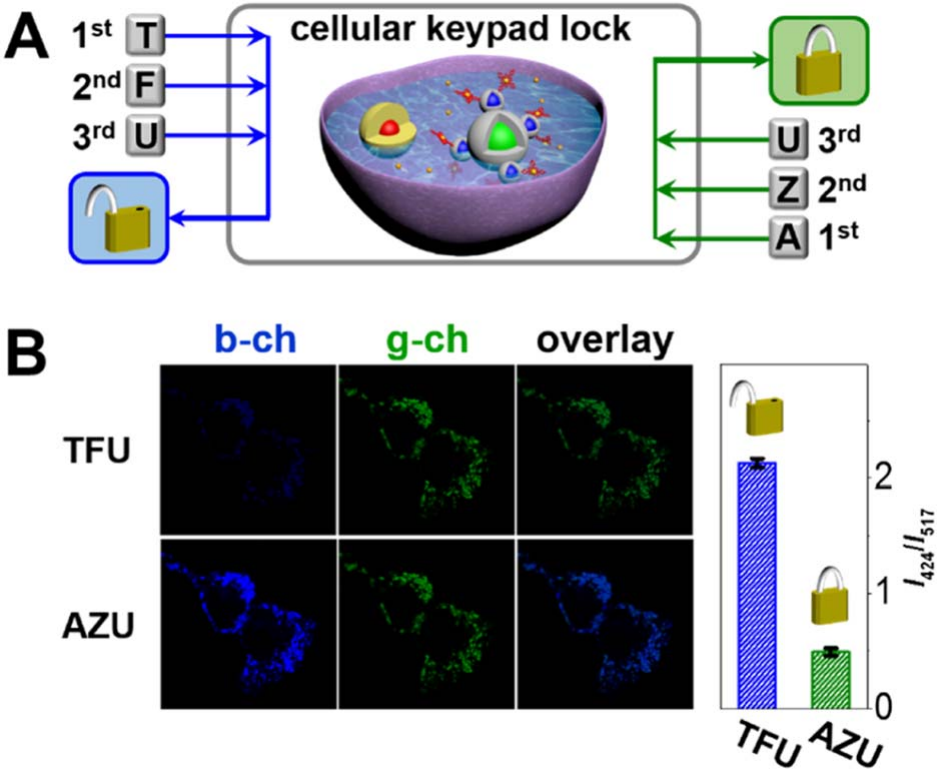
(A) Computation of the cellular keypad lock. (B) Fluorescent (blue-channel and green-channel) images of the cellular keypad lock for the TFU and AZU input combinations. Scale bar: 10 μm. Histograms show blue-to-green emission intensity ratios of the encrypted and decrypted cellular keypad lock.

Inspired by the logic relationships between extracellular stimulations and intracellular responses, we sought to develop a cellular keypad lock. This is particularly attractive because it represents a new approach to information protection at the single-cell level (Fig. 6A). The cellular keypad lock was constructed by transplanting nanodevices into mammalian cells *via* co-incubation. The nanodevice was found to heavily enter the cells within 4 h (Fig. S10 in the ESI†). Afterwards, the cells were fixed in order to study the logical response of an individual cell. In the unlock and lock operations, the cellular keypad locks were subjected to sequential chemical and optical inputs: TFU and AZU. As shown in Fig. 6B, the green-channel fluorescence remains stable, while the blue-channel fluorescence varies with respect to the state of the cellular keypad lock. Quantitatively, the blue-to-green intensity ratios are 2.2 and 0.5 for the input combinations of TFU and AZU, respectively. As a result, the overlaid cell images display distinct fluorescent colours: green and blue indicate encrypted and decrypted cellular keypad locks, respectively. In this bio-encryption system, the nanodevice mimics a subcellular component that can process extracellular chemical or optical inputs and output an optical signal according to the keypad lock arithmetic.

## Conclusions

In conclusion, we have constructed a robust QD–TSPP FRET nanodevice and implemented 3-input keypad lock operations for the first time. The chemical input combination of the keypad lock provides competitive chelation reactions that can change the chelation state of TSPP in the correct order. The fluorescent output of the keypad lock is controlled by the absorption wavelength shift of TSPP-induced QD–TSPP FRET at the nanoscale. The fluorescence of the keypad lock is outputted in a ratiometric manner, which offers high signal readability *via* distinguished colour changes. Both unlock and lock operations follow the 3-input keypad lock arithmetic with high resettability. Beyond the operations in homogenous solution, the nanodevice can be used to facilely construct paper and cellular keypad locks, respectively, showing high potential in personal information identification and bio-encryption applications. Overall, this QD-FRET nanodevice based keypad lock paves up a low-cost but robust way toward next-generation security devices.

## Author contributions

Peng Shen: investigation, data curation, formal analysis, visualization, and writing – original draft. Yuqian Liu: investigation, formal analysis, resources, and funding acquisition. Xiaojun Qu: methodology and validation. Mingsong Zhu: investigation, and formal analysis. Ting Huang: visualization. Qingjiang Sun: conception, supervision, project administration, funding acquisition, and writing – review & editing.

## Conflicts of interest

There are no conflicts to declare.

## Supplementary Material

NA-005-D3NA00030C-s001
