# Supplementary material for: An optical keypad lock with high resettability based on a quantum dot–porphyrin FRET nanodevice
Source: Nanoscale Adv. 2023 May 8;5(11):2986–93. doi: 10.1039/d3na00030c (PMC10228340; doi:10.1039/d3na00030c)
Supplement: NA-005-D3NA00030C-s001 [file NA-005-D3NA00030C-s001.pdf]

Electronic Supplementary Material (ESI) for Nanoscale Advances.
This journal is © The Royal Society of Chemistry 2023

Electronic Supplementary Information

An Optical Keypad Lock with High Resettability Based on A QD-TSPP FRET Nanodevice

Peng Shen,†[a] Yuqian Liu,†[b] Xiaojun Qu,[a] Mingsong Zhu,[a] Ting Huang,[a] and Qingjiang Sun*[a]

†Authors contribute equally.

[a] State Key Laboratory of Bioelectronics, School of Biological Science & Medical Engineering, Southeast University, Nanjing 210096, China

[b] Jiangsu Co-Innovation Center of Efficient Processing and Utilization of Forest Resources and Joint International Research Lab of Lignocellulosic Functional Materials, College of Light Industry and Food Engineering, Nanjing Forestry University, Nanjing 210037, China

Email: sunqj@seu.edu.cn (Qingjiang Sun)

Supplementary Experimental Section

Synthesis of APTMS-TSPP

To construct bQD@SiO_2-TSPP, modification of TSPP with 3-(trimethoxysilyl)-1-propanamine (APTMS) according to the reported method.[1] Briefly, 0.05 mmol TSPP and 0.026 mmol $SOCl_2$ were added to 60 mL of toluene and refluxed for 8 h in Ar atmosphere. After cooling to ambient temperature, the toluene was removed by rotary evaporation, and the chlorinated TSPP (TSPP-Cl) was moved into the glovebox for further use. The linkage of TSPP and APTMS was performed by adding 0.02 mmol of TSPP-Cl and an excess amount of APTMS (0.05 mmol) in 10 mL of anhydrous DMF. The solution was persistently stirred for 8 h, followed by the removal of DMF and purification of TSPP-APTMS with a silica column. The eluent was $CHCl_3$:CH_3OH (20:1), which can effectively separate TSPP-APTMS and other components. The optical properties were characterized for confirming the successful preparations of TSPP-Cl and TSPP-APTMS. For TSPP-Cl, three absorption peaks arise at 550, 596, and 648 nm, and is non-fluorescent. For TSPP-APTMS, the absorption peaks locate at 515, 555, 587, and 633 nm, and the fluorescence peak shows at 721 nm.

Preparations of gQD@SiO_2, bQD@SiO_2-TSPP and gQD@SiO_2/bQD@SiO_2-TSPP nanodevice

Oil soluble gQDs (CdSe/ZnS, EM: 517 nm) and bQDs ($Cd_xZn_{1-x}S$/ZnS, EM: 424 nm) were synthesized following the reported protocols.[2,3] The gQD@SiO_2 was prepared by a modified microemulsion method.[4] The positive-charged bQD@SiO_2 was prepared by ligand exchange with (3-mercaptopropyl)trimethoxysilane (MPTMS), followed by amination with APTMS. The ligand exchange was conducted in the glovebox for preventing the hydrolysis of MPTMS in the presence of water. In detail, 2 μL of MPTMS and 1 mg of bQDs were mixed in toluene (1 mL). The oleic acid on surface of bQDs was replaced by MPTMS after 4 h of vigorous stirring. The amination was performed by adding 120 μL of APTMS solution (100 mM in toluene) into the MPTMS-modified bQDs solution. Hydrolysis of MPTMS and APTMS was initiated by moving the flask to the ambient condition for allowing the entrance of moisture. After 12 h of stirring, the bQD@SiO_2 was isolated by centrifugation (10000 rpm, 12 min). By mixing TSPP-APTMS with APTMS during the hydrolysis process, the bQD@SiO_2-TSPP was facilely prepared. For optimizing the TSPP density, molar ratio of APTMS and TSPP-APTMS was tuned from 1:0 to 0:1, and the fluorescence quenching degree of bQDs was measured. The gQD@SiO_2 and bQD@SiO_2-TSPP were dispersed in PBS buffer (10 mM, pH 7.0) for further use. The gQD@SiO_2/bQD@SiO_2-TSPP nanodevice was assembled by dropwise adding 0.5 mL gQD@SiO_2 solution (0.05 μM) into 1 mL of QD@SiO_2-TSPP solution (0.5 μM). After 10 min of stirring, the nanodevice was precipitated by centrifugation (3000 rpm, 5 min), and was dispersed in 1mL of PBS buffer.

Metalation and transmetalation of TSPP

All reagents were dissolved in PBS buffer (10 mM, pH 7.0) for metalation and transmetalation of TSPP. Especially, $FeSO_4{\cdot}7H_2O$ was dissolved in the buffer degassed by Ar_2. 1 μM TSPP solution was firstly mixed with 1.5 μM $ZnCl_2$ solution or 1.5 uM $FeCl_3$ solution in equal volumes to prepare Zn(II)TSPP or Fe(III)TSPP, respectively. Under the catalysis of imidazole (60 mM)[5] or $FeSO_4{\cdot}7H_2O$ (1.5 uM),[6] the standing time for Zn(II)TSPP or Fe(III)TSPP were 30 min and 60 min, respectively. For the experiment of Zn(II) to Fe(III) transmetalation of TSPP, 0.5 μM of Zn(II)TSPP solution was first mixed with an equal volume of TPEN (4 μM) solution, after 40 min standing, 10 μM $FeCl_3$ (containing 1 Equiv. of $FeSO_4{\cdot}7H_2O$) was added into the TPEN treated Zn(II)TSPP, the incubation time was 60 min. A similar procedure was processed for Fe(III) to Zn(II) transmetalation, while 4 μM ASC was used as the demetallation reagent of Fe(III)TSPP with an incubation time of 30 min. Formation of Zn(II)TSPP started with adding 10 μM $ZnCl_2$ into the mixture under the catalysis of 60 mM imidazole. The transformation of Zn(II)TSPP-TSPP-Fe(III)TSPP was monitored by scanning the soret-band (360-450 nm) absorptions of the porphyrins.

Characterizations

The zeta potential measurements of gQD@SiO_2, bQD@SiO_2-TSPP, as well as the nanodevice, were conducted in PBS buffer (10 mM, pH 7.0) with a Malvern Zetasizer Nano-ZS particle analyzer (Malvern, U.K.). Transmission electron microscopy (TEM) images of the nanoparticles and nanodevice were acquired on a JEOL JEM-2100 instrument (JOEL, Japan). The compositional analysis of powder samples of gQD@SiO_2/bQD@SiO_2 and gQD@SiO_2/bQD@SiO_2-TSPP was performed on a JSM-7600F scanning electron microscopy (SEM, Hitachi, Japan) equipped with energy dispersive X-ray spectroscope (EDX). The atom concentrations of Si, N, and S were derived from the comparisons with standard samples of SiO_2, BN, and FeS_2, respectively. Fourier transform-infrared (FT-IR) measurements were conducted with dry samples of gQD@SiO_2, bQD@SiO_2(-TSPP), or nanodevice on Nicolet-5700 instrument (Nikon, Japan). The UV-vis absorption spectra of TSPP treated with metal ions or metal chelators were measured with a Hitachi U-4100 spectrophotometer (Hitachi, Japan). All fluorescence measurements were performed on a Hitachi F-4600 fluorescence spectrophotometer (Hitachi,

Japan), with solutions placed in a 0.2 cm quartz cuvette. The excitation wavelength was fixed at 405 nm with a slit width of 5 nm. Fluorescence images of cellular encryption devices were obtained from TCS SP8 confocal fluorescence microscope (Leica, Germany). The excitation wavelength of the laser was 405 nm and emissions were collected at 410 nm–430 nm and 510 nm–530 nm.

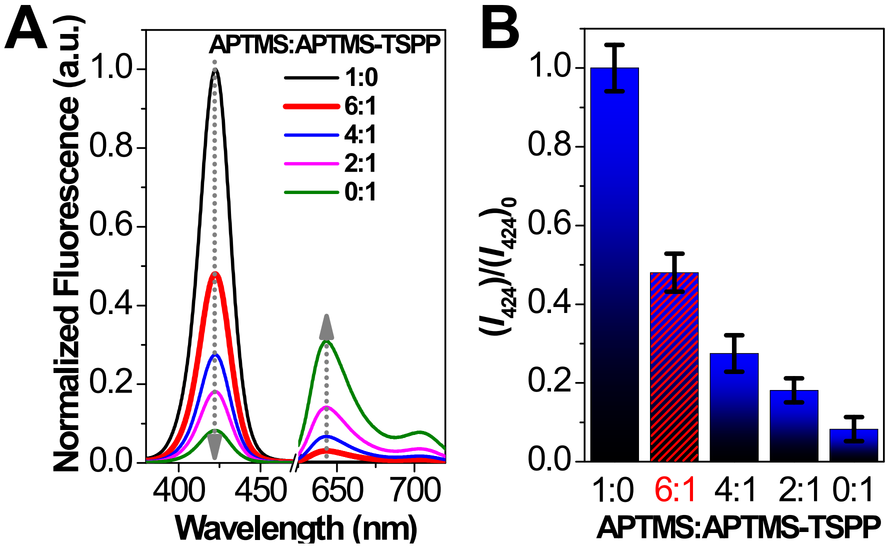

Figure S1. (A) Evolution of the fluorescence spectra, and (B) the relative fluorescence intensity of bQD@SiO_2-TSPP with varying TSPP densities.

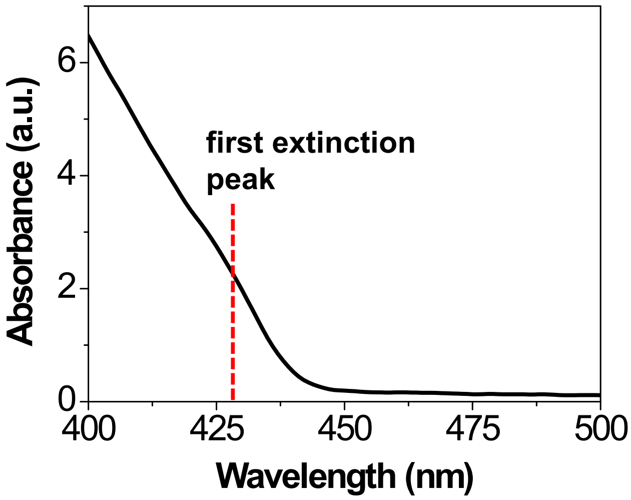

Figure S2. Absorption spectra of bQDs solution (1 mg in 30 mL of chloroform) for estimation of its molar concentration with the Lambert–Beer's law ($A = \varepsilon CL$). In the equation, ***A*** represents the absorption intensity at first extinction peak (428 nm). ε is the extinction coefficient of QDs with CdSe core, which is calculated to be 6.17×10^5 $M^{-1}cm^{-1}$ according to the equations reported by Peng et al.[7] ***L*** is the path length of the cuvette (1 cm). The calculated concentration and amount of bQDs are 3.18 μM and 9.54×10^{-8} mol, respectively. The optimized APTMS-TSPP amount is (molar ratio of APTMS to APTMS-TSPP is 6:1) calculated as 1.71×10^{-7} mol. Therefore, the stoichiometry of TSPP to bQDs is determined as averagely 1.8 TSPP molecules are modified on each bQD@SiO_2 particle.

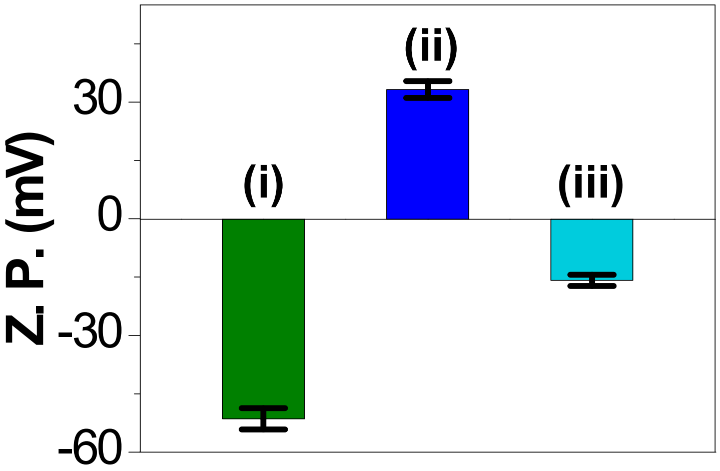

Figure S3. Zeta potentials of (i) gQD@SiO_2, (ii) bQD@SiO_2-TSPP and (iii) gQD@SiO_2/bQD@SiO_2-TSPP.

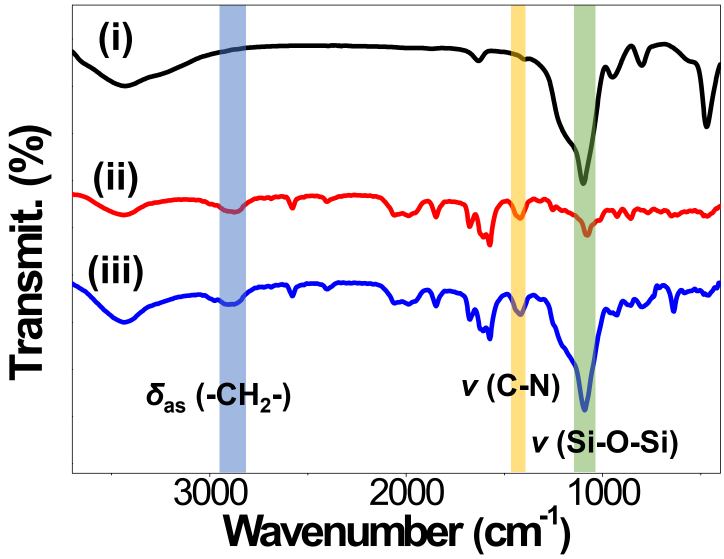

Figure S4. FT-IR spectra of (i) gQD@SiO_2, (ii) bQD@SiO_2-TSPP and (iii) gQD@SiO_2/bQD@SiO_2-TSPP. Shadows in the figure indicate the deformation vibrations of -CH_2- linker at 2890 cm^{-1} introduced by APTMS (blue shadow), stretching vibration of C-N bond in TSPP (yellow shadow, 1419 cm^{-1}), and the stretching vibration of Si-O-Si (green shadow, 1095 cm^{-1}) in silica. In comparison with the spepctrum of gQD@SiO_2, emergence of the deformation vibrations of -CH_2- implies the assembling of bQD@SiO_2, which is prepared by the use of sailane agents with alkane chains (MPTMS and APTMS). Further, the appearance of stretching vibration of C-N bond on the spectrum of gQD@SiO_2/bQD@SiO_2-TSPP indicates the successful modification of TSPP, which contains aromatic N-heterocycle.

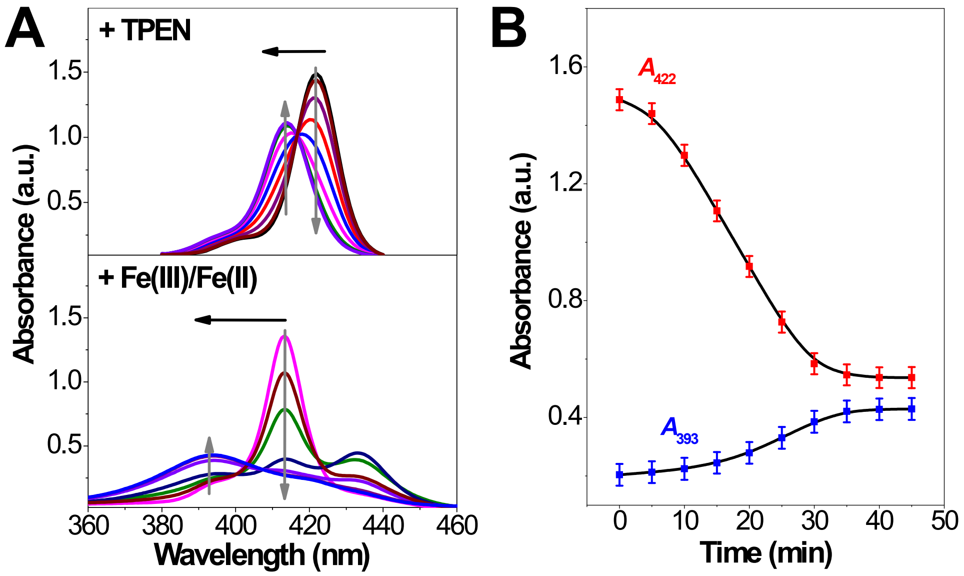

Figure S5. (A) Successive blue-shift of the absorption spectra of TSPP in the Zn(II) to Fe(III) transmetalation process upon treating with TPEN andiron ions. (B) Evolutions of the maximum absorption of Zn(II)TSPP ($\boldsymbol{A}_{422}$) and Fe(III)TSPP ($\boldsymbol{A}_{393}$) with respect to time.

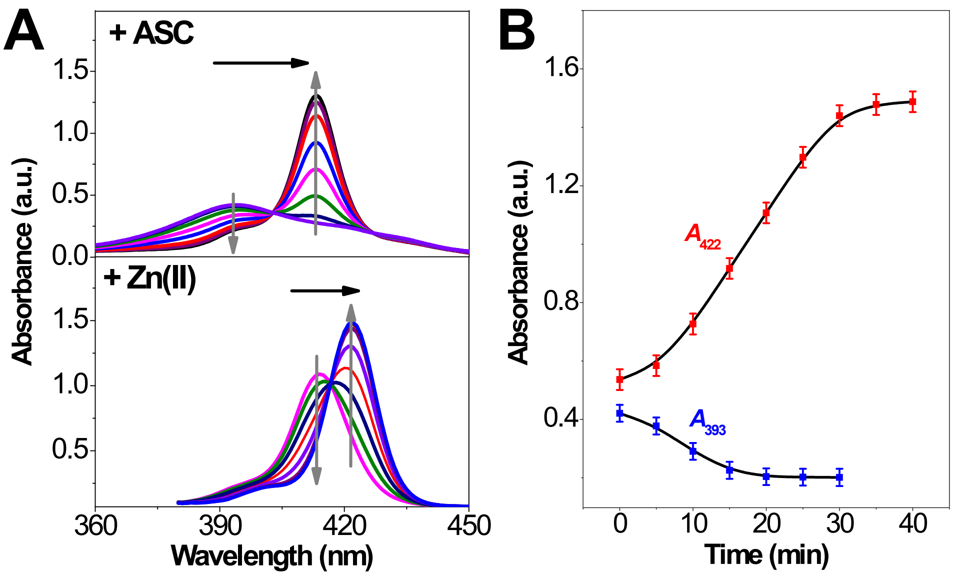

Figure S6. (A) Successive red-shift of the absorption spectra of TSPP in the Fe(III) to Zn(II) transmetalation process upon treating with ASC and Zn(II). (B) Evolutions of the maximum absorption of Zn(II)TSPP ($\boldsymbol{A}_{422}$) and Fe(III)TSPP ($\boldsymbol{A}_{393}$) with respect to time.

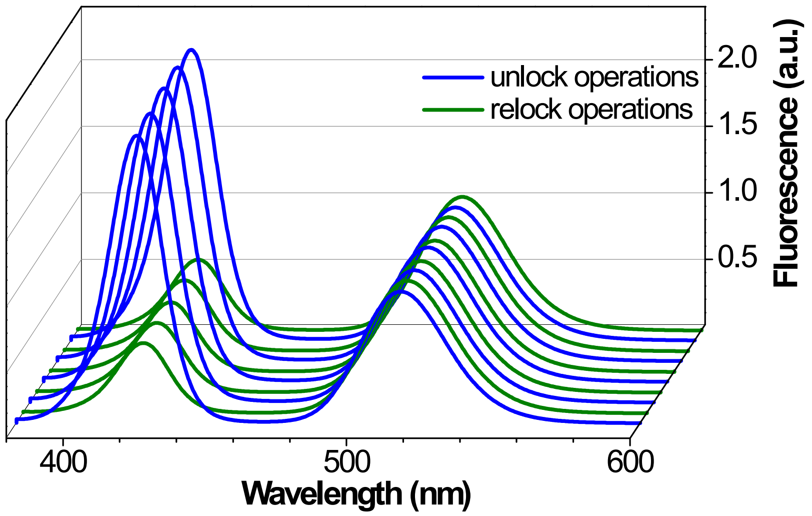

Figure S7. Fluorescence spectra of the nanodevice for 5 repeated unlock and lock operations.

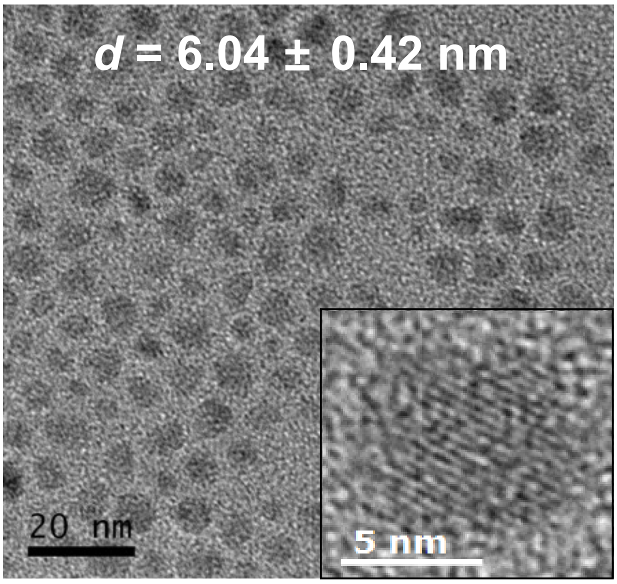

Figure S8. TEM image and high resolution TEM image of bQDs. The average diameter (***d***) is 6.04 nm by counting 50 particles.

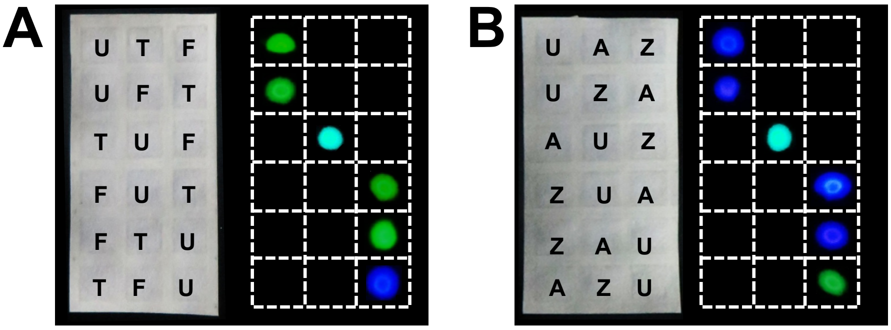

Figure S9. Fluorescent images of the paper keypad lock for varying input combinations in the (A) unlock and (B) lock operations. The images were taken while the UV light was introducing by an UV laser (365 nm).

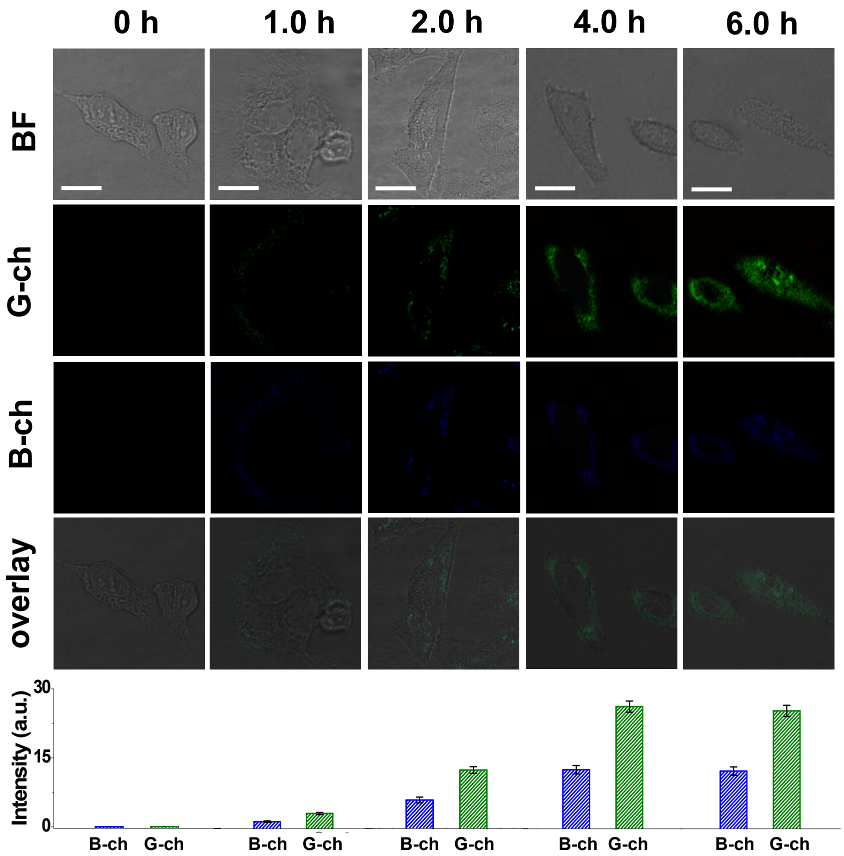

Figure S10. Fluorescent images (green channel and blue channel) of HeLa cells treated with the nanodevice for 0-6 h. Scale bar: 1 μm. The histograms represent the fluorescence intensities of green and blue channels of the cytoplasm, which are calculated with ImageJ.

Supporting references

1. S. Farhadi, A. Zabardasti and M. H. Rahmati, *J. Chem. Res.*, 2011, **3**, 157–160.
2. W. K. Bae, K. Char, H. Hur and S. Lee, *Chem. Mater.*, 2008, **20**, 531–539.
3. K. H. Lee, J. H. Lee, W. S. Song, H. Ko, C. Lee, J. H. Lee and H. Yang, *ACS Nano*, 2013, **7**, 7295–7302.
4. T. Aubert, S. J. Soenen, D. Wassmuth, M. Cirillo, R. Van Deun, K. Braeckmans and Z. Hens, *ACS Appl. Mater. Interfaces*, 2014, **6**, 11714–11723.
5. H. Ishii and H. Tsuchiai, *Anal. Sci.*, 1987, **3**, 229–233.
6. R. Huszank, G. Lendvay and O. Horvath, *J. Biol. Inorg. Chem.*, 2007, **12**, 681–690.
7. W. W. Yu, L. Qu, W. Guo, and X. G. Peng, *Chem. Mater.*, 2003, **15**, 2854-2860.
